# Deciphering the molecular crosstalk between type 2 diabetes and pancreatic cancer through cross-disease co-expression network analysis

**DOI:** 10.1016/j.bbrep.2026.102562

**Published:** 2026-03-31

**Authors:** Fariba Dehghanian, Sheyda Khalilian, Zaynab Mousavian, Shahryar Alavi, Amin Bahreini

**Affiliations:** a Department of Cell and Molecular Biology and Microbiology, Faculty of Biological Science and Technology, University of Isfahan, HezarJarib Street, Isfahan, 81746-73441, Iran; b Division of Infectious Diseases, Department of Medicine Solna, Karolinska Institute, Stockholm, Sweden; c School of Mathematics, Statistics and Computer Science, College of Science, University of Tehran, Tehran, Iran; d Palindrome, Isfahan, Iran; e Poursina Hakim Digestive Diseases Research Center, Isfahan University of Medical Sciences, Iran

**Keywords:** Pancreatic cancer, Type 2 diabetes mellitus, Co expression network, WGCNA, *ACADVL* gene

## Abstract

Multiple complex mechanisms link type 2 diabetes mellitus (T2DM) with the pathogenesis, development, and progression of pancreatic cancer (PC). This study aims to elucidate these complex relationships using cross-disease co-expression analysis of PC and T2DM. Transcriptomic data from peripheral blood samples of patients with pancreatic ductal adenocarcinoma (PDAC), PDAC patients with diabetes (DP), patients with diabetes mellitus (DM), and healthy controls were analyzed. Following differential expression analysis (DEA), four disease-specific gene co-expression networks were constructed using weighted gene co-expression network analysis (WGCNA). Pearson correlation analysis was then applied to identify modules significantly associated with each clinical trait.

In the experimental phase, peripheral blood samples from 20 PDAC patients, 20 DP patients, 20 DM patients, and 20 healthy controls were included. The co-expression network analysis identified modules highly associated with PDAC, DP, and DM. Among the 11 overlapping genes shared between these modules, the high-confidence hub genes *ACADVL*, *AGTRAP*, and *PADI4* were selected for quantitative real-time PCR (qPCR) validation. Comparative analysis of *ACADVL* expression among the four study groups showed significantly higher expression in the PDAC group than in the DM group (p < 0.01) and healthy controls (p < 0.0001). Similarly, *ACADVL* expression was significantly elevated in the DP group compared with the DM group (p < 0.01) and healthy controls (p < 0.0001). The survival analysis also suggests that high *ACADVL* expression is a favorable prognostic biomarker.

## Highlights

• Shared gene co-expression links pancreatic cancer and type 2 diabetes (T2D).
• Integrated RNA-seq and WGCNA yield 11 candidate markers for PDAC and T2D.
• ACADVL, AGTRAP, and PADI4 emerge as key genes in PDAC-T2D comorbidity.
• Differential and co-expression analyses highlight a major role for ACADVL.

## 1 Introduction

PC has a poor prognosis and is the seventh leading cause of cancer-related death worldwide. Due to lifestyle changes, PC was expected to overtake breast cancer as the third greatest cause of cancer-related death by 2025. PDAC is the most common type of PC (>90%) and has a low 5-year overall survival rate (9%) [[1], [2], [3]]. Molecular targeted therapy has not resulted in a significantly improved prognosis despite advances in understanding PDAC biology. As a result, researchers have steadily focused on its prevention and diagnosis, including efforts to prevent transformed cells from becoming malignant. Based on this, a better understanding of the risk factors underlying PDAC development is critical for identifying and developing preventive and intervention strategies [4]. At the time of diagnosis, 45-65% of patients with PDAC have diabetes mellitus (DM), with approximately 25% having new-onset diabetes. On the other hand, there is evidence that DM increases the risk of PDAC by 2-fold [5]. A recent study showed diabetic patients with adjuvant therapy and PC resection had larger tumors and a higher death rate than non-diabetic patients [6]. Lee et al. reported that the early recurrence rate in PC patients after resection primarily correlates with new-onset DM, suggesting that new-onset DM may be an important clinical indicator of PC and that detecting it may help early diagnosis [7].

Recently, a significant number of studies have focused on deciphering the underlying molecular mechanisms of diabetic PDAC. Patients with DM exhibit insulin resistance, elevated insulin-like growth factor-1 (IGF-1) levels, and hyperinsulinemia, all of which are potential growth-promoting factors [4]. Insulin and IGF-1 activate the PI3K signaling pathway by binding to their respective receptors. These receptors are shown to be expressed in human pancreatic cancer cells [8]. T2DM is characterized as a chronic, inflammatory condition characterized by elevated levels of reactive oxygen species and proinflammatory cytokines. This inflammatory environment may promote tumor genesis and progression [4,9,10]. There is an urgent need to identify reliable biomarkers for early PDAC diagnosis and monitoring. Bioinformatics linked with microarray technology has shown promise in investigating the molecular mechanisms of malignant cancers in recent decades. The weighted gene co-expression network analysis (WGCNA) is a holistic method for classifying highly related genes in gene expression data into modules and connecting each module to sample traits [11,12]. In recent years, WGCNA has been widely used across different applications, including the discovery of potential biomarkers in various cancers [[13], [14], [15], [16]].

This study constructed a weighted gene co-expression network and screened novel blood-based biomarkers of diabetic PDAC. First, the differentially expressed genes between different groups were identified. Then, the module-trait relationship was used to determine the candidate module associated with diabetic PDAC. Finally, among the 11 overlapping genes between these modules, the high-scoring hub genes *ACADVL*, *AGTRAP,* and *PADI4* were selected for qPCR validation in 20 patients with PDAC, 20 with DP, 20 with DM, and 20 healthy controls.

## 2 Materials and methods

### 2.1 Data acquisition and preprocessing

**Fig. 1:**
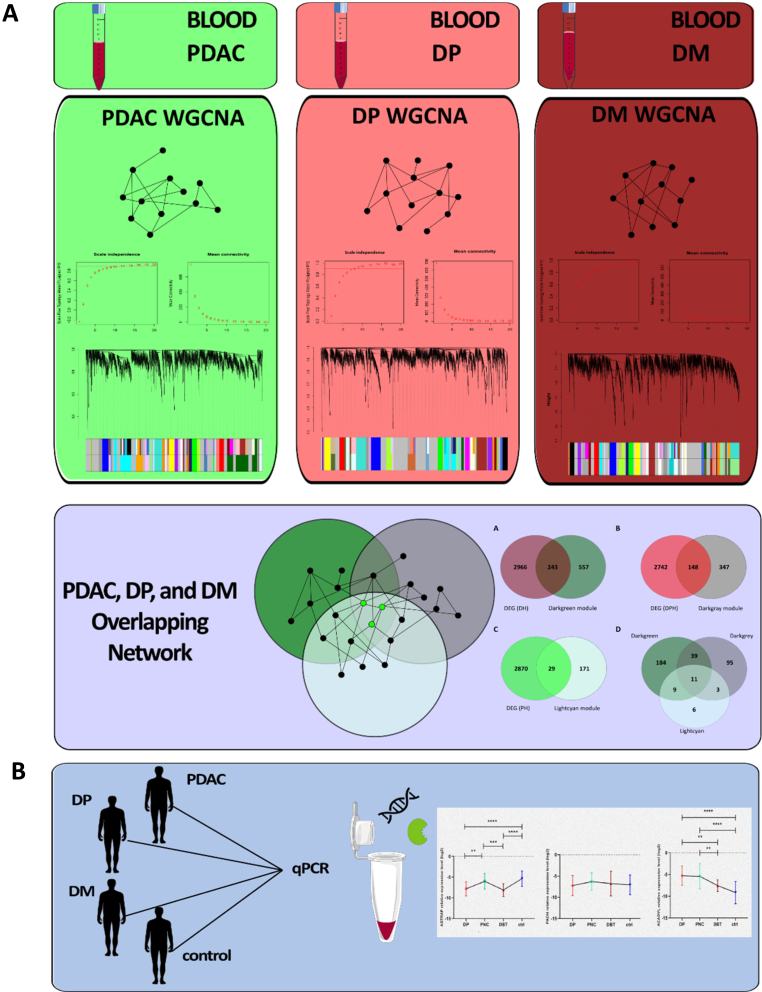
**Schematic workflow of the study.** (A) Transcriptomic data from blood samples of DM, DP, and PDAC patients and healthy individuals were extracted. Co-expression network analysis was then performed to identify the most critical modules for each disease relative to the control. (B) Finally, Peripheral blood samples were obtained from healthy controls, DM, DP, and PDAC patients to validate the role of the most critical genes suggested through in silico analyses.

A schematic workflow of the study is represented in Fig. 1. The microarray dataset GSE15932 comprises 32 peripheral blood samples, including eight PDAC patients, eight PDAC patients with diabetes (DP), eight patients with diabetes mellitus (> five years) (DM), and eight healthy controls [17]. This dataset was generated using the Affymetrix Human Genome U133 Plus 2.0 Array platform, and the original Affymetrix CEL files were downloaded from the GEO database (http://www.ncbi.nlm.nih.gov/geo) [18]. All CEL files were preprocessed with the oligo package (version 3.6) in R 3.4.1 using the Robust Multi-chip Analysis (RMA) function for background adjustment, normalization, and summarization [19]. Multiple probes mapping the same gene symbol were merged using the CollapseRow function (MaxMean method) [20].

### 2.2 Differential expression analysis

After preprocessing, 20421 genes were used for differential expression analysis (DEA). The DEA was performed between PDAC and control samples, DP and control samples, and DM and control samples using the limma (Linear Models for Microarray Analysis) R package [21]. We used the |log2 fold change| ≥ 0.58 and the *p* value < 0.05 to identify significantly differentially expressed genes in each comparison.

### 2.3 Construction of Co-expression networks

We used the WGCNA R package to construct a gene co-expression network and cluster the network into modules to identify the most relevant module for each trait of interest. For each PDAC, DM, and DP trait, we used both healthy control and disease samples to construct the network. Before network construction, hierarchical clustering of samples was performed using the flashClust function in R to identify outliers [12,22]. In WGCNA, the pickSoftThreshold function was used to select the optimal power value, resulting in a co-expression network with a more scale-free topology. After network construction for each trait, all network genes were clustered into modules using a hierarchical clustering algorithm, with each module colored distinctively. All genes that can't be assigned to any module were placed in the grey module, which was ignored in further analysis. Finally, the mergeCloseModules function was used to merge highly correlated modules (cutHeight = 0.25) [23].

### 2.4 Relating Co-expression modules to external clinical traits

The Pearson correlation between each module eigengene and the trait vector was calculated to identify which modules are significantly associated with each trait of interest. To define the trait vector for each PDAC, DM, and DP trait, we assigned 0 to healthy control samples and 1 to disease samples. For each module, the first principal component is defined as the module eigengene, representing the gene expression variation within that module. Both Module Membership (MM), the association of a gene to each module, and Gene Significance (GS), the correlation between a gene and the trait vector, can be used to detect the most essential genes in each module. Genes with high MM and GS values for all three traits (PDAC, DP, DM) were identified for subsequent analysis. Then, the clusterProfiler package in R was used to identify the candidate module's significantly associated GO and KEGG pathways (adjusted p-values <0.05).

### 2.5 Study population

Peripheral blood samples were obtained from healthy controls, diabetic patients, and patients pathologically diagnosed with PDAC who underwent Whipple surgery at Milad or Zahraye Marzieh Hospital, Isfahan, Iran. Informed consent from the patients was obtained before sample collection. Finally, 20 patients diagnosed with PDAC, 20 PDAC patients with diabetes mellitus, 20 diabetic patients, and 20 healthy controls were included. The Demographic and clinicopathological characteristics of the participants were provided in Supplementary Table 1. The study was approved by the University of Isfahan Regional Research Ethics Committee (IR.UI.REC.1398.082). Peripheral blood mononuclear cells (PBMC) were isolated by Ficoll (Lymphodex, InnoTrain, Germany) gradient separation and frozen until analysis.

### 2.6 RNA extraction, cDNA synthesis, and quantitative real-time PCR

Total mRNA from PBMC samples was extracted using RiboEX (GeneAll Biotechnology) according to the manufacturer's standard protocol. The quality and quantity of RNA samples were assessed using a NanoDrop One/OneC spectrophotometer (Thermo Scientific). RNA was reverse-transcribed into cDNA using the AddScript cDNA Synthesis Kit (Addbio, Korea) according to the manufacturer's instructions. Finally, RT-qPCR was performed using the RealQ Plus Master Mix Green (Amplicon, Denmark) and the StepOne Real-time PCR system (Applied Biosystems). The primer sequences used in RT-qPCR are presented in Supplementary Table 2. *BACT* was used as an endogenous control.

### 2.7 Statistical analysis

Analysis of variance (ANOVA) was used to examine differences between groups. P < 0.05 was considered statistically significant. All calculations were performed by GraphPad Prism version 13.0. The GEPIA2 database, which queries the TCGA pancreatic adenocarcinoma (PAAD) cohort, was used for survival analysis (https://gepia3.bioinfoliu.com/).

## 3 Results

### 3.1 Differential expression analysis and Co-expression network construction

**Fig. 2:**
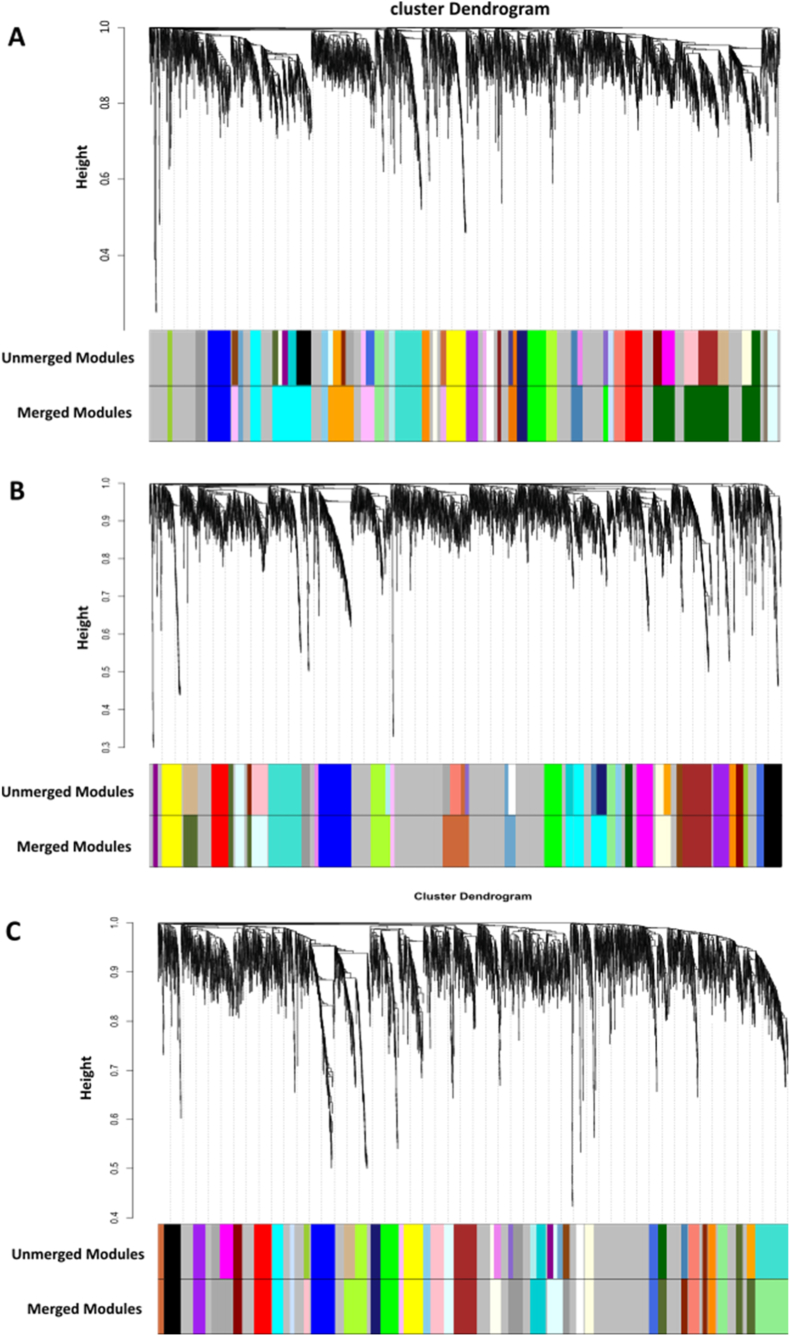
**The module eigengene clustering and hierarchical clustering dendrograms of DM (A), PDAC (B), and DP (C) modules.** The dendrogram was built based on a dissimilarity measure (1-TOM). Each colored bar indicates a specific module that contains highly co-expressed genes. MEDissThres 0.25 was used to merge highly correlated modules.

We obtained a normalized expression matrix for the GSE15932 dataset, containing eight PDAC patients, eight PDAC patients with diabetes (DP), eight patients with diabetes mellitus (>5 years) (DM), and eight healthy controls. Based on the screening criteria as |log2FC| ≥ 0.58 and *p* value < 0.05, 133, 341, and 392 DEGs were identified in PDAC-control (PH), DP-control (DPH), and DM-control (DH), respectively. Based on the pickSoftThreshold function of the WGCNA package, a soft-thresholding power of 10 for DH, 8 for DPH, and 12 for PH (Supplementary Fig. 1) was used for network construction to obtain a scale-free network. Afterward, WGCNA gene clustering was used to split genes into modules based on the topological overlap matrix. Finally, a 0.25 cutoff was used to merge highly correlated modules using the mergeCloseModules function. 29, 31, and 30 modules were discovered in PDAC-control, DP-control, and DM-control, respectively (Fig. 2).

### 3.2 Module-trait relationship analysis indicates that lightcyan, darkgrey, and darkgreen

**Fig. 3:**
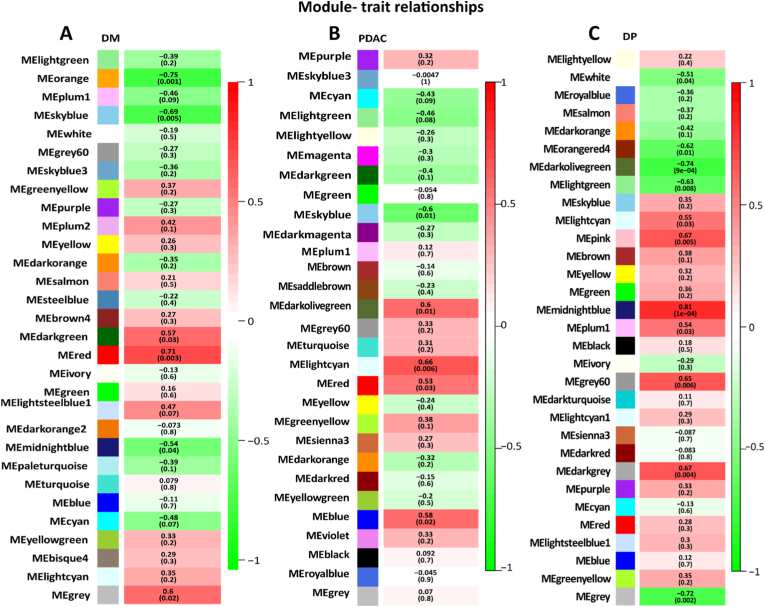
**Heatmap of the correlation (from -1 to +1) between module eigengenes and the DM (A), PDAC (B), and DP (C) phenotypes, with p-values.** Red and darkgreen modules are highly related to DM phenotypes; lightcyan module is highly related to PDAC; and midnightblue and darkgray modules are highly associated with DP.

Module eigengene is defined as the first principal component of the module's gene expression pattern. The relationship between each module and trait was discovered using Pearson's correlation between the module eigengene and the module trait (PDAC, DP, and DM). Lightcyan module is highly related to PDAC; midnightblue and drakgray modules are highly associated with DP; and red and darkgreen modules are highly related to the DM phenotype (Fig. 3). A Venn diagram was used to discover the overlap between the significant modules of each network. The lightcyan, darkgrey, and darkgreen modules have the most overlapping genes among the PDAC, DP, and DM networks. GO enrichment analyses were performed to assess the function of co-expressed genes in the lightcyan, darkgrey, and darkgreen modules. As represented in Supplementary Figs. 2A, B, and C, lightcyan, darkgrey, and darkgreen modules are enriched in myeloid and leucocyte activation, leucocyte migration, and actin filament polymerization and organization.

**Fig. 4:**
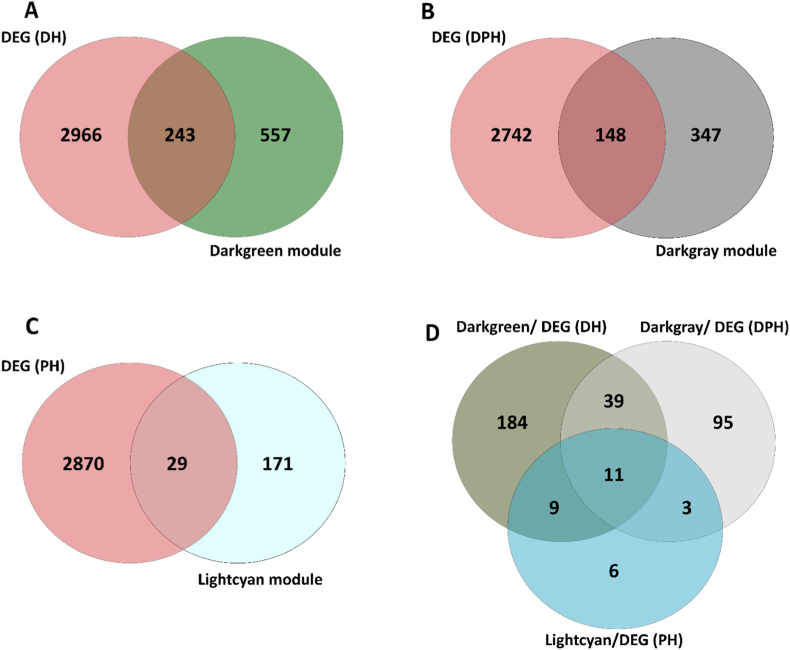
**The Venn diagram shows the overlap between differentially expressed genes and members of the significant modules in DM (A), DP (B), and PDAC (C).** The Venn diagram of A, B, and C's common genes shows eleven genes highly associated with DM, PDAC, and PD (D).

In the next step, a venn diagram was used to find the overlap genes between the lightcyan, darkgrey, and darkgreen members with PDAC, DP, and DM DEGs, respectively (Fig. 4A, B, and C). Finally, a Venn diagram was used to identify the 11 overlap genes between DEGs and critical modules (Fig. 4D). Among the 11 overlapping genes (Supplementary Table 3) between these modules, the high-scoring hub genes *ACADVL*, *AGTRAP*, and *PADI4* were selected for qPCR validation. All these three genes were upregulated based on |log2 fold change| ≥ 0.58 and the *p*-value <0.05 criteria.

### 3.3 Assessment of the relative gene expression levels of *ACADVL*, *AGTRAP*, and *PADI4* genes in PDAC, DP, DM, and control groups

**Fig. 5:**
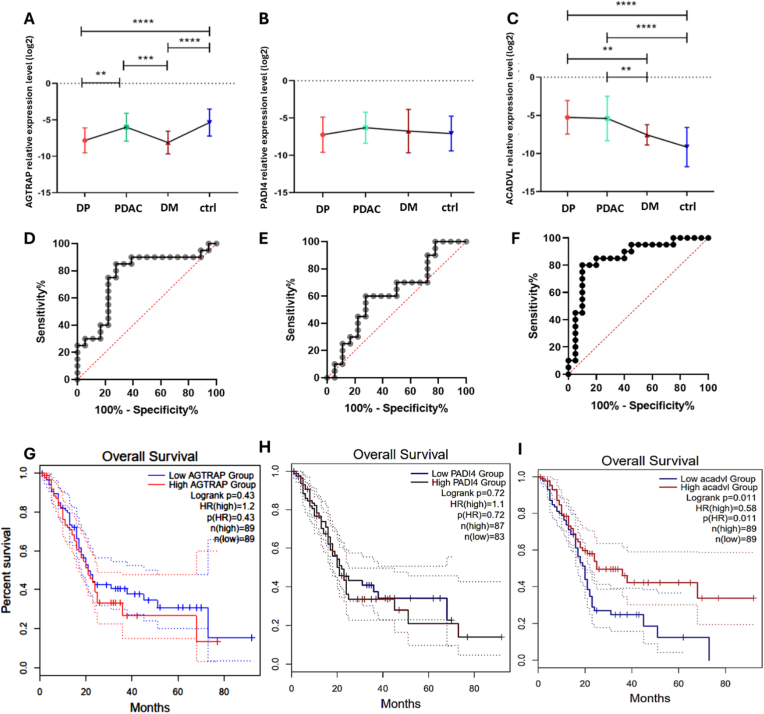
**Expression profiles, diagnostic capacity, and prognostic value of AGTRAP, PADI4, and ACADVL**. (A–C) Analysis of the relative expression levels of *AGTRAP* (A), *PADI4* (B), and *ACADVL* genes across four different clinical cohorts: DP, PDAC, DM, and healthy controls (ctrl). The data points represent the mean expression, and error bars indicate the standard deviation (or standard error). Statistical significance between groups is denoted by asterisks (**p < 0.01, ***p < 0.001, ****p < 0.0001). (D–F) ROC curves evaluating the diagnostic sensitivity and specificity of *AGTRAP* (AUC: 0.76, *p* value: 0.005) (D), *PADI4* (AUC: 0.62, *p* value: 0.19) (E), and *ACADVL* (AUC: 0.85, *p* value: 0.0001) (F). The red dotted diagonal line represents the line of no-discrimination (random guess). (G–I) Kaplan-Meier overall survival (OS) curves for patients, stratified into “High” (red line) and “Low” (blue line) expression groups for *AGTRAP* (G), *PADI4* (H), and *ACADVL* (I). The Log-rank test was used to determine statistical significance. Hazard ratios (HR), p-values, and the number of patients in each group (n) are indicated within the respective plots. Dotted lines representing the confidence intervals are also shown for each survival curve.

For the *AGTRAP* gene, the relative expression comparison among the four groups showed increased expression in the PDAC group compared to the DP (p-value <0.01) and DM (p-value <0.001) groups. *AGTRAP* was downregulated in the DP group (*p*-value <0.0001) and DM group (*p*-value <0.0001) compared to the control group (Fig. 5A). *PADI4* expression did not differ significantly between PDAC, DP, DM, and control samples (Fig. 5B). A comparison of *ACADVL* expression across the four study groups showed increased expression in the PDAC group compared to the DM group (*p*-value <0.01) and the control group (*p*-value <0.0001). Also, *ACDVL* was overexpressed in the DP group compared to DM (*p*-value <0.01) and the control group (*p*-value <0.0001) (Fig. 5C).

Diagnostic test evaluation was performed for the AGTRAP (Fig. 5D), PADI4 (Fig. 5E), and ACADVL (Fig. 5F) genes, which showed significant overexpression in the PDAC, DP, and DM groups compared with the control, as indicated by ROC curves and AUC calculations. According to the ACADVL expression level in the PDAC versus the control group, the AUC was 0.85 (95% CI 0.73–0.98; *p* value: 0.0005). To investigate the clinical relevance of all three genes, we performed survival analysis using the GEPIA2 database, which queries the TCGA pancreatic adenocarcinoma (PAAD) cohort. For *the AGTRAP (*Fig. 5G*) and PADI4 (*Fig. 5H*) genes, the survival curves of the high- and low-*expression groups cross each other and are very close. This indicates that the expression levels of the AGTRAP and PADI4 genes do not significantly affect patients' overall survival. It cannot be used as a reliable prognostic marker. Interestingly, our analysis revealed that high ACADVL expression is significantly associated with prolonged overall survival. As shown in Fig. 5I, patients with high ACADVL levels had a better prognosis compared to those with low expression (Log-rank P = [0.011]; Hazard Ratio [HR] = [0.58]).

## 4 Discussion

PDAC remains one of the deadliest malignancies worldwide, with a five-year survival rate below 5%. The absence of early symptoms makes timely diagnosis extremely difficult, underscoring the urgent need to identify reliable biomarkers and better understand the molecular mechanisms driving PDAC onset and progression [24]. A well-established clinical observation is that approximately 80% of PDAC patients also have T2D, suggesting a meaningful biological link between the two conditions [25,26]. T2D not only increases the risk of developing PDAC but is also associated with larger tumor size and higher mortality compared to non-diabetic patients [5,6]. Several mechanisms have been proposed to explain this relationship, including elevated insulin and IGF-1 levels activating the PI3K signaling pathway in pancreatic cancer cells, as well as chronic accumulation of reactive oxygen species (ROS) and pro-inflammatory cytokines, which promote tumor progression [4,6,[8], [9], [10]]. Despite these insights, reliable biomarkers shared between DM and PDAC remain largely undefined. To address this gap, we applied a systems biology framework combining differential expression analysis with WGCNA to identify hub genes shared across PDAC, DP, and DM diseases. It identifies genes not merely based on individual expression changes, but also on coordinated co-expression patterns across multiple phenotypes. This is particularly important in our study, where we aimed to find genes whose dysregulation is shared across PDAC, DP, and DM, a question that standard DEG analysis is not designed to answer efficiently. WGCNA enabled us to identify biologically coherent, phenotype-associated modules, providing a systems-level rationale for candidate gene selection that DEG alone cannot offer. From 133, 341, and 392 genes significantly dysregulated in PDAC, DP, and DM samples, respectively, we identified three overlapping hub genes, including *ACADVL*, *AGTRAP*, and *PADI4* genes, and validated their expression by qPCR.

*ACADVL* is associated with GO terms related to fatty acid beta-oxidation, mitochondrial function, and lipid metabolic processes, consistent with known metabolic reprogramming in PDAC*.* [27]. While prior studies have associated *ACADVL* downregulation with cervical tumors and adrenocortical carcinomas [28,29], our findings reveal a contrasting pattern: *ACADVL* was significantly overexpressed in blood leukocytes of PDAC patients. This overexpression was confirmed by both qPCR and ROC curve analysis, suggesting its potential as a non-invasive diagnostic biomarker. Notably, *ACADVL* expression was highest in PDAC compared to DM and controls. Also, it was elevated in DP relative to DM, pointing to a progressive upregulation tied to cancer rather than metabolic disease alone. Our survival analysis further showed that higher *ACADVL* expression correlates with longer patient survival, positioning it as a favorable prognostic biomarker. This is a clinically important finding that warrants validation in larger, independent cohorts. These results are partially consistent with growing evidence that mitochondrial metabolic reprogramming plays a key role in pancreatic cancer. However, our finding of overexpression rather than downregulation distinguishes PDAC from other tumor types, highlighting the context-dependent role of *ACADVL* across cancer subtypes.

*AGTRAP* has recently emerged as a candidate biomarker in several cancers, including lower-grade glioma, colon, tongue, and hepatocellular carcinoma, where its expression correlates with poor prognosis. AGTRAP is linked to GO terms involving angiotensin receptor binding, regulation of the MAPK cascade, and cellular response to hormone stimulus. [[30], [31], [32], [33]]. In our study, bioinformatic analysis predicted *AGTRAP* upregulation; however, qPCR results showed significant downregulation in DP and DM blood samples, with no significant change in PDAC. Taken together, our data suggest that *AGTRAP* downregulation may be a feature of T2D conditions rather than PDAC, warranting further mechanistic investigation.

*PADI4* has been reported to be overexpressed across multiple tumor types, including gastric, breast, esophageal, lung, and pancreatic cancers. PADI4 is associated with GO terms related to citrullination, chromatin remodeling, and immune response regulation. [[34], [35], [36], [37]]. In particular, Chang et al. demonstrated PADI4 overexpression in blood samples from patients with pancreatic and several other cancers [37]. Contrary to these reports, we did not observe significant *PADI4* dysregulation in our cohort. This discrepancy may be attributable to differences in sample composition, patient ethnicity, disease stage, or the specific tissue compartment analyzed (tumor vs. blood). This highlights the need for standardized multi-cohort studies when evaluating *PADI4* as a pan-cancer biomarker.

Our study shares conceptual overlap with Zhou et al. (2021), who also applied WGCNA to identify DP-related modules using PDAC datasets (GSE74629 and GSE15932) [38]. A key distinction of our work is the simultaneous comparison of three disease groups, including PDAC, DP, and DM, against healthy controls, enabling us to dissect condition-specific versus shared gene expression patterns. Furthermore, our inclusion of qPCR validation strengthens the translational relevance of the identified hub genes. While Zhou et al. focused on dataset-level verification, our approach bridges computational discovery with experimental confirmation in patient-derived samples.

This discrepancy between computational predictions and experimental validation for the *AGTRAP* and *PADI4* genes may reflect differences in patient cohort characteristics (age, disease stage, ethnicity, comorbidities), sample sizes, normalization strategies across GEO datasets and our own samples, and inherent batch effects between microarray and qPCR quantification platforms. On the other hand, regarding the stage-dependent expression, the GEO datasets used may not have distinguished disease stage, potentially masking stage-specific expression. Our sample size may also have been insufficient to detect modest expression changes in a highly variable gene. Several important questions remain open. First, the diagnostic and prognostic value of *ACADVL* should be tested in larger, multi-center cohorts stratified by disease stage and diabetes status. Second, functional studies are needed to determine whether *ACADVL* plays a causal role in PDAC progression or serves primarily as a metabolic bystander. Third, the mechanistic basis for *AGTRAP* downregulation in T2D conditions and its potential interaction with insulin signaling pathways deserves dedicated investigation. Finally, integrating multi-omics data (e.g., proteomics, metabolomics) with the co-expression networks identified here could yield a more comprehensive picture of the T2D–PDAC molecular interface and accelerate the development of combination diagnostic panels.

## 5 Conclusion

Using an integrated bioinformatic approach, we identified *ACADVL* as a promising blood-based biomarker for PDAC diagnosis and prognosis, with supporting qPCR and survival data. Our comparative analysis across PDAC, DP, and DM groups provides a systems-level view of shared and distinct gene expression changes, contributing new insights into the molecular overlap between T2D and PDAC. Validation in larger, independent cohorts and functional characterization of the identified hub genes represent essential next steps toward clinical translation.

## Authors contribution

FD contributed to project design, interpretation, manuscript writing, and figure preparation. SK contributed to performing the experiments. ZM contributed to performing data analysis and interpretation. SA contributed to data analysis. AB contributed to the collection of patient samples.

## Ethics statements

Approval of the research protocol by an Institutional Review Board: To the principles outlined in the Declaration of Helsinki and performed with permission by the responsible Ethics Committee of the University of Isfahan. Informed Consent: All patients were informed of the study's purposes and have consequently signed their consent forms. Registry and the Registration No. of the study/trial: IR.UI.REC.1398.082. Animal Studies: N/A.

## Funding information

This work was supported by grants from the financial support of the grant received from the Research Vice-Chancellor of the 10.13039/501100007087University of Isfahan.

## Declaration of competing interest

The authors declare that they have no conflict of interest.

## Data Availability

The data that has been used is confidential.
