# Supplementary material for: Deciphering the molecular crosstalk between type 2 diabetes and pancreatic cancer through cross-disease co-expression network analysis
Source: Biochem Biophys Rep. 2026 Mar 31;46:102562. doi: 10.1016/j.bbrep.2026.102562 (PMC13068620; doi:10.1016/j.bbrep.2026.102562)
Supplement: Multimedia component 1 [file mmc1.docx]

|  | **Control**  **(n=20)** | **PDAC**  **(n=20)** | **T2D**  **(n=20)** | **PDAC-T2D**  **(n=20)** |
| --- | --- | --- | --- | --- |
| **Age (Year)** | 32-67 | 33-77 | 36-67 | 31-79 |
| **Male/Female** | 9/11 | 9/11 | 11/9 | 10/10 |
| **Serum CA_19-9_ (U/ML)** | 28 ± 7 | 428.87±120.6 | - | 501.75±113.5 |
| **Metastasis** |  | 4/20 |  | 6/25 |
| **T2D Treatment (Yes / No)** | - | - | 20/0 | 20/0 |
| **Type of T2D Treatment** | - |  | Metformin (10), Insulin (7), Other (3) | Metformin (11), Insulin (6), Other (3) |
| **Diabetes duration** | - | - | > 5 years | > 5 years |

**Supplementary table 1.** **Demographic and clinicopathological characteristics of the** **participants.**

***Unit/Milliliter**

| **Primer** | **Sequence** |
| --- | --- |
| ACADVL-F | 5`-CCTTTGCAACACCCAGTACG-3` |
| ACADVL-R | 5`-GCAGGATGCCTTTGAAACCG-3` |
| AGTRAP-F | 5`-CTCCATCGACGCCATAAGCA-3` |
| AGTRAP-R | 5`-TTGAGCAGCAAGCTGAGGAT-3` |
| PADI4-F | 5`-TTGATCCGTGTGACCCCAGA-3` |
| PADI4-R | 5`-TTTCTTCTTGGCTGGAGGGC-3` |
| ACTB-F | 5`-TTCGAGCAAGAGATGGCCA-3` |
| ACTB-R | 5`-CACAGGACTCCATGCCCAG-3` |

**Supplementary table 2**: Primer sequences used for real-time quantitative reverse transcriptase.

**Supplementary table 3:** The list of 11 overlapping genes among PDAC, DP, and DM groups.

**logFC**: Log Fold Change; **AveExpr**: Average Expression; **t**: t-statistic; **P.Value**: P-Value; **B**: B-statistic (Log-Odds)
