# Supplementary material for: Deciphering the molecular crosstalk between type 2 diabetes and pancreatic cancer through cross-disease co-expression network analysis
Source: Biochem Biophys Rep. 2026 Mar 31;46:102562. doi: 10.1016/j.bbrep.2026.102562 (PMC13068620; doi:10.1016/j.bbrep.2026.102562)
Supplement: Multimedia component 2 [file mmc2.docx]

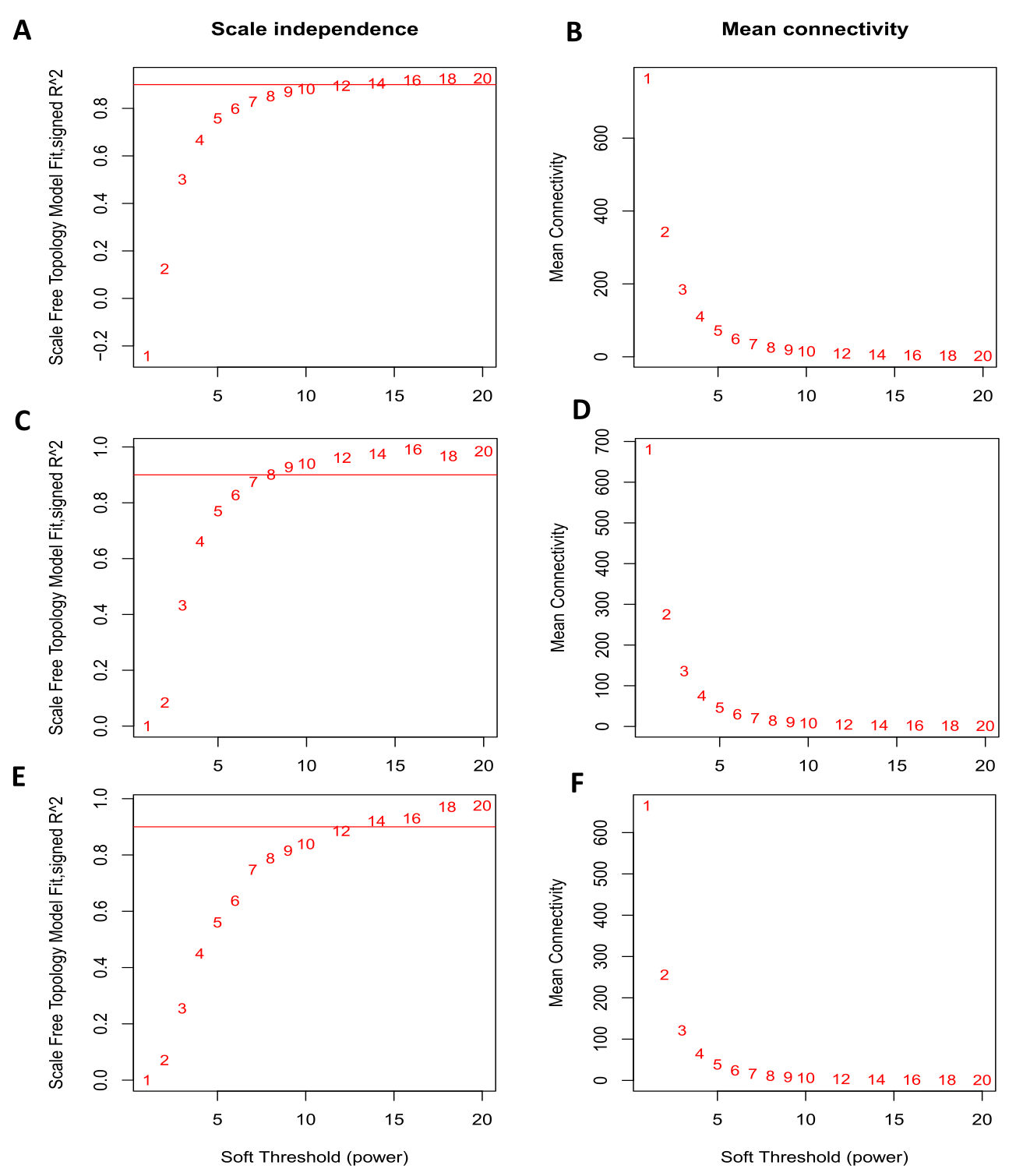
**Supplementary figure 1. Determination of soft-thresholding power in WGCNA.** Analysis of the scale-free fit index for various soft-thresholding powers (β) and analysis of the mean connectivity for various soft-thresholding powers in (A,B) DH, (C, D) DPH, (E, F) PH respectively.


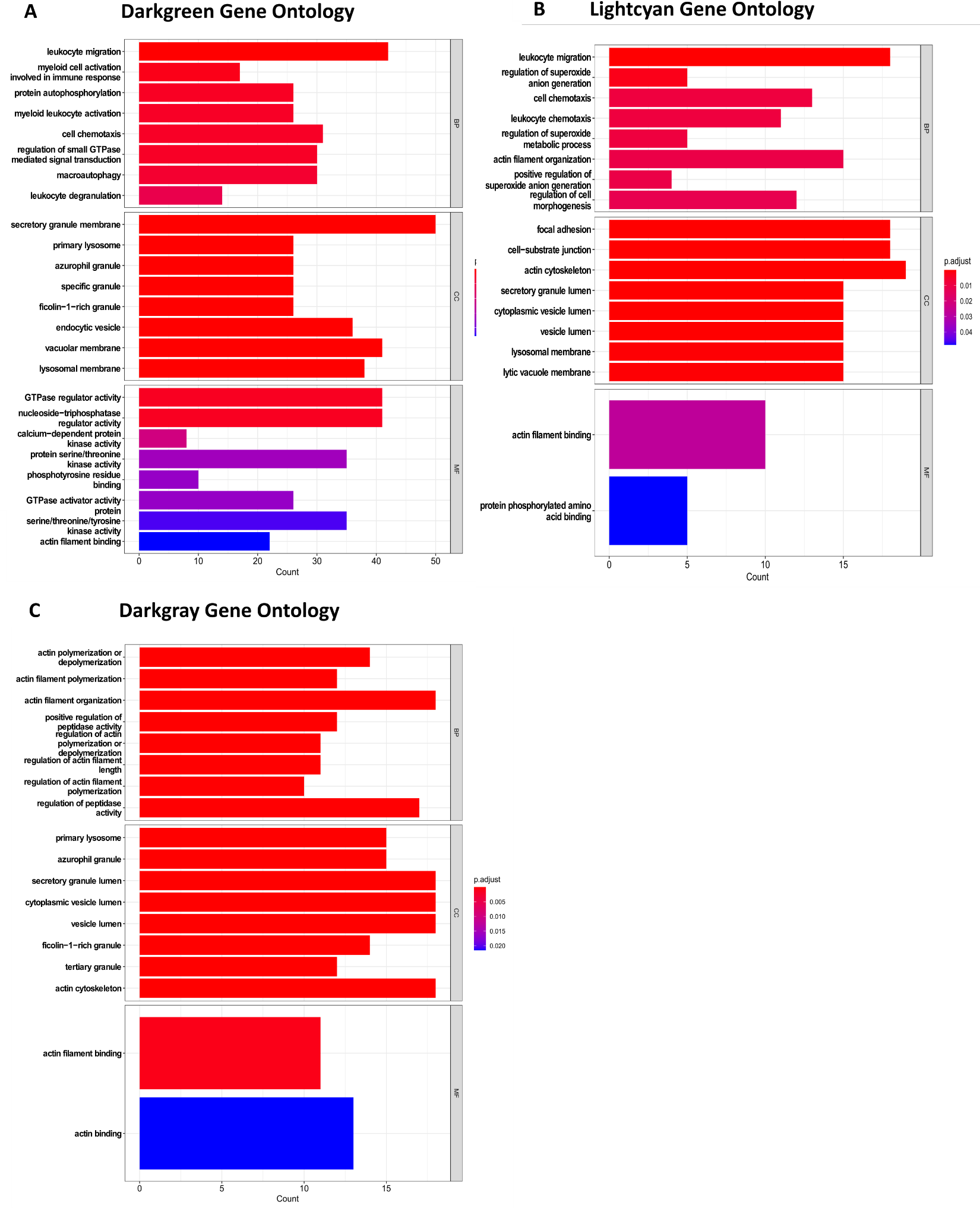


**Supplementary figure 2.** GO term enrichment analysis results for (A) darkgreen module, (B) lightcyan module, and (C) darkgrey module. GO functional enrichment analyses of genes in the candidate modules, including darkgreen, lightcyan, and darkgrey, suggest specific roles in leucocyte activation, leucocyte migration, and actin filament polymerization and organization. The number of genes is shown on the x-axis, and GO terms are shown on the y-axis. The bar plot shows the biological process (BP), molecular function (MF), and their respective cellular components (CC) for the GO terms. The adjusted p-values for each term are colored according to the legend.
